# Influence of Cavity Lining on the 3-Year Clinical Outcome of Posterior Composite Restorations: A Randomized Controlled Clinical Trial

**DOI:** 10.3390/dj12050128

**Published:** 2024-05-07

**Authors:** Anh Duc Nguyen, Natalie Pütz, Mary Michaelis, Kerstin Bitter, Christian Ralf Gernhardt

**Affiliations:** University Outpatient Clinic for Conservative Dentistry and Periodontology, Department of Dentistry, Medical Faculty, Martin-Luther-University Halle-Wittenberg, Magdeburger Strasse 16, 06112 Halle, Germanykerstin.bitter@uk-halle.de (K.B.)

**Keywords:** nanohybrid composite, flowable composite materials, clinical study, direct restoration, randomized clinical trial

## Abstract

The purpose of this randomized, split-mouth-designed controlled and single-blinded clinical study was to evaluate the 3-year clinical performance of Class I and Class II resin composite restorations placed with or without cavity lining with a flowable composite. Fifty patients with treatment needs in two premolars or molars were included. One of the teeth was restored using the nanohybrid composite (Grandio^®^SO, control group), in the test group a high viscosity flowable composite was additionally applied as a first layer. In both groups, the same self-etch adhesive system was applied. Clinical evaluation after 3 years was carried out using the modified USPHS/Ryge criteria. At the 3-year follow-up the recall rate was 92%. Four restorations failed in the test group (8.7%), three due to the loss of vitality and one after fracture. The control group exhibited a cumulative success rate of 100%, while the test group achieved a success rate of 91.3%. This led to significant differences in the annual failure rate (AFR) between the two groups, with rates of 0% and 2.9% (*p* < 0.05; Mann–Whitney U-test). After 3 years the cumulative survival rate including all restorations was 95.7%. Statistical analysis revealed significant differences for the parameters: tooth vitality, marginal discoloration, success rate, and AFR. The other parameters exhibited no significant differences. Consequently, the nanohybrid composite demonstrated excellent performance over a 3-year period, whereas the utilization of a flowable composite for the cavity lining did not appear to exert a beneficial influence on clinical outcomes.

## 1. Introduction

As is known from numerous publications in the field of adhesive and aesthetic dentistry, direct composite restorations have become an accepted and widely used alternative to other directly usable materials or indirect restoration procedures using laboratory- or CAD/CAM-fabricated restorations made of metal or ceramic materials in posterior teeth [1,2,3,4]. Today, direct composite restorations showed comparable long-term results to other indirect restoration possibilities [2,5,6,7,8]. Furthermore, their use fits in the actually recommended aesthetic, tooth-saving, minimally invasive, and cost-effective therapeutic concept [9]. Regarding clinical success in restorative dentistry, marginal adaptation or marginal leakage followed by secondary caries or postoperative hypersensitivity are the most common clinically observable complications over time, in particular in deep dentinal-bonded approximal lesions [10,11,12]. Furthermore, a clinically very difficult to detect phenomenon is inadequate internal bonding of the adhesive materials to tooth hard tissues resulting in insufficient internal and marginal adaption [13]. This problem is often described to be correlated with the occurrence of postoperative sensitivity, restoration loss, and finally caries [14]. Therefore, several publications recommended the supplementary application of a flowable composite for the cavity lining to reduce polymerization stress and improve adaption of the adhesive filling materials [13,15]. Their conclusion was that the main intention of this clinical protocol is to avoid prospective complications such as microleakage and the above-mentioned subsequent clinical problems for our patients [16,17]. However, focusing on the clinical effect of this protocol using flowable composites as cavity liners concerning all important and already mentioned consequences, the clinical impact on longevity of composite restorations remain unclear and is controversial in the literature [6,18,19,20,21,22]. However, despite the scientific discussion regarding the clinical value of a lining protocol, there is an observable ongoing increased use of flowable resins as a cavity lining and stress-relieving increment during the placement of composite restorations in general dentistry [17,23,24,25,26]. Seemann et al. published the results of a survey in Germany which found that 80.1% of the participating dentists use a flowable composite for cavity lining [27]. Compared to most of the used flowable composite materials for this procedure the flowable resin material (Grandio^®^SO Heavy Flow, VOCO GmbH, Cuxhaven, Germany) used in our clinical long-term study is a so-called heavy flowable nanohybrid composite with a relatively high viscosity compared to other flowable materials [28,29].

Many investigations evaluating the influence of lining with flowable composites on the clinical long-term performance of direct composite restorations [5,29,30,31] reported favorable results. However, valid clinical data and evidence, particularly regarding a longer observation time than 24 months, especially for the high-viscosity flowable composite used, which might be more effective when used as a liner in the posterior teeth requiring increased strength [28], are still rare [29,32]. The aim of the present paper, following the study protocol developed and approved in advance [29] and examining the clinical outcome of the described material and material combinations over an observation period of several years, was to report the results after 3 years.

The primary goal outlined in the study protocol, as presented in the 24-month results, was to assess the efficacy of incorporating a flowable composite as a lining material alongside a nanohybrid composite for the restoration of Class I and II cavities over a period of 3 years.

The secondary goal was to evaluate the clinical outcome and behavior of the used nanohybrid composite material regarding different parameters over the observation period of 3 years. The third goal was to examine if the used self-etch adhesive system is suitable for providing a long-term success of the restorations over 3 years.

## 2. Materials and Methods

The comprehensive study protocol of this clinical trial is included in the previously published interim report [29]. However, for better readability of the work, parts of the methodology are also described in the current publication focusing on the 3-year results.

### 2.1. General Study Design

**Figure 1:**
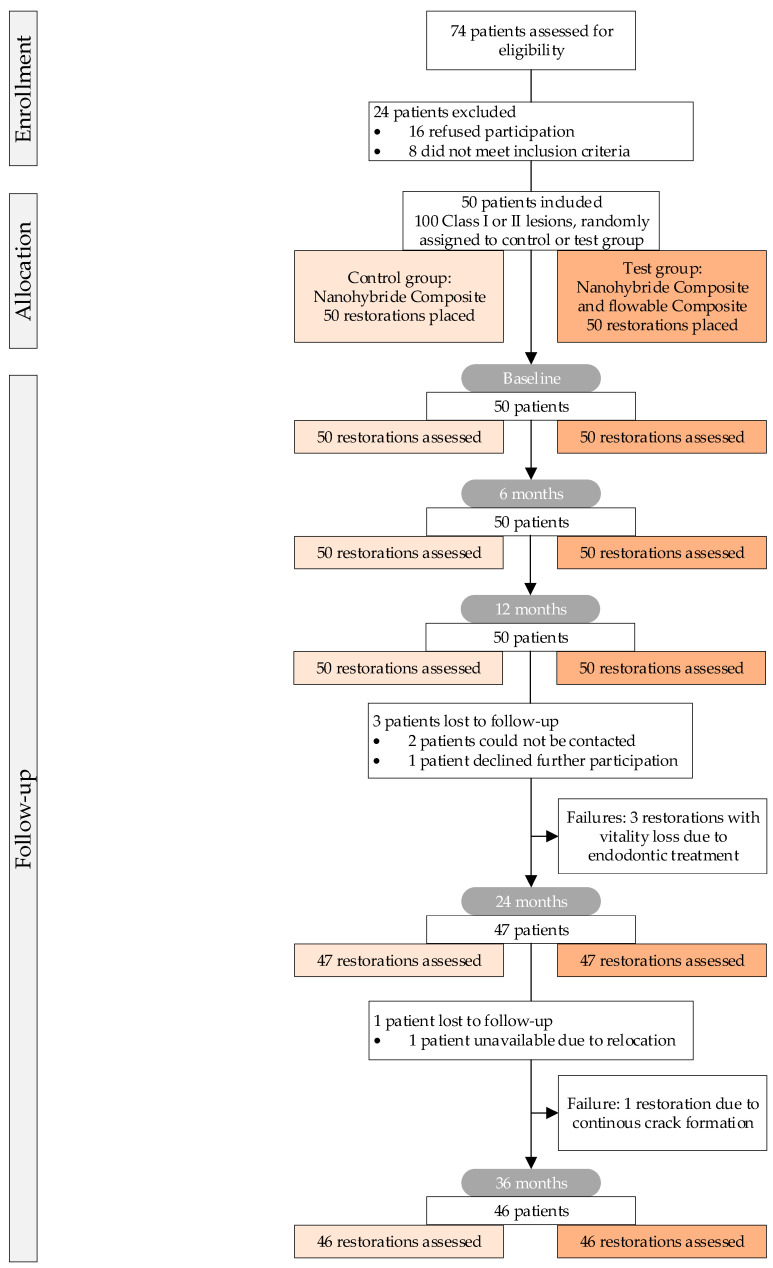
CONSORT Flow Chart: Enrollment, allocation, and follow-up representing the relevant appointments and information.

Initially, 74 patients exhibiting restorative treatment needs in a minimum of two teeth underwent screening. Following the prospective study protocol, fifty patients who met the inclusion criteria (Table 1) having at least two posterior teeth (premolars or molars) with treatment needs for direct composite restorations (Figure 1) were included. The study protocol received approval from the Ethics Committee of the Medical Faculty of Martin Luther University Halle-Wittenberg, Halle, Germany (protocol number: 225/01.12.10/8). The clinical study was registered at the German Clinical Trials Register (DRKS), the German WHO primary registry, located at the Federal Institute for Drugs and Medical Devices (Germany, registration number or DRKS-ID: DRKS00033585).

Screened participants were extensively informed and additionally received written information about the entire study in advance, and signed the consent forms indicating their willingness to be part of the investigation. Teeth with profound carious lesions that underwent indirect pulp capping during the restorative process were also included, following the study protocol. Given the split-mouth design of the clinical investigation, two defects of nearly comparable size were allocated at random to the two different treatment groups (control and test group) using a randomization list provided by the statistician for each patient. Throughout the trial, all clinical procedures, as per the study protocol, were conducted by a single experienced dentist (Figure 1).

### 2.2. Clinical Process

At the beginning, a thorough medical history, dental, and radiographic examination were performed to assess restorative needs, coronal, and apical pathologies, following the same procedure as reported in the 24-month results [29]. Restorative treatment in all cases was carried out after local anesthesia and adequate rubber dam isolation of the affected and selected teeth using the nanohybrid composite. In class-II cavities, an additional matrix system was used. The two restorations on both sides following the split-mouth design were placed in one session. In the test group, an additional layer of flowable composite (Grandio^®^SO Heavy Flow) was applied on the cavity floor (maximal increment of 0.5 mm) using a dental explorer. If needed, after removing inadequate extensive fillings and deep caries, punctual indirect pulp capping was performed using a calcium-hydroxide-containing liner (Calcicur, VOCO GmbH, Cuxhaven, Germany). 

In both groups, following cavity preparation, a self-etch adhesive system (Futurabond^®^ DC, VOCO GmbH, Cuxhaven, Germany) was scrubbed into the surface for a minimum of 20 s using the provided brushing device, commencing on enamel. The material was spread to a thin layer using compressed air and polymerized for 10 s (Celalux^®^ 2, VOCO GmbH, Cuxhaven, Germany, approximately 1300 mW/cm^2^, wavelength range 450–480 nm). Restorations were layered in increments of a maximum thickness of 2 mm and polymerized for 30 s. Finally, the surfaces of the restorations were finished using differently grained diamonds (Komet, Gebr. Brasseler GmbH & Co. KG, Lemgo, Germany) and polished.

### 2.3. Re-Examniation Protocol (Baseline, 6 Months, 12 Months, 24 Months, 36 Months)

The clinical re-examinations were done by a second blinded, experienced, and calibrated dentist who was not involved in the restoration procedure. A baseline examination was performed two weeks after the restorative procedure. Follow-up appointments were performed after 6, 12, 24, and 36 months. The modified USPHS/Ryge criteria were used for clinical assessment of the restorations in both groups (Table 2) [33,34,35]. Furthermore, different oral hygiene parameters were used to determine the quality of individual oral hygiene, the impact of the applied instructions and periodontal health: the gingival index (GI) and proximal plaque index (PI) proposed by Silness and Löe [36,37].

### 2.4. Statistical Considerations

In cooperation with the Institute for Medical Epidemiology, Biometrics and Informatics, Interdisciplinary Centre for Health Sciences at the Martin Luther University Halle-Wittenberg, sample size calculation was performed in advance, prior to ethical approval. This calculation was based on the expectation that the defined primary endpoint, the annual failure rate (AFR), would be 1.5% in the test group and 2.1% in the control group, combined with an assumed standard deviation of 1. Detecting clinically relevant differences with 80% power at a 5% significance level required the inclusion of 44 patients (with 44 restorations per arm in a split-mouth design). Taking a potential moderate dropout rate into account, a total of 50 participants (representing 50 composite restorations per arm) should be finally included [29]. All statistical analysis were conducted using SPSS^®^ 25.0 (IBM^®^, Ehningen, Germany). Mann–Whitney U-test was employed at a 5% level of significance to detect statistically significant differences between the investigated groups.

## 3. Results

### 3.1. Demographic and Clinical Characteristics of the Study Population at Baseline

The gender distribution within the study population was homogeneous. After screening 74 patients, 29 women and 21 men were included in the study (Figure 1). In addition to the randomization that was conducted, the process was determined by the indication of the teeth to be treated. At baseline, a total of 42 premolars and 58 molars were treated. Furthermore, 22 of the included 42 premolars and 28 of the 58 molars were assigned to the control group (Figure 1). The observable distribution within the test and control group was not significantly different. A total of 32 Class I and 68 Class II lesions were restored and evaluated. The distribution within the control and test groups was comparable (Table 3).

### 3.2. Development of the Study Population over 36 Months

After 36 months of observation, 46 patients out of 50 (28 female, 18 male) could be re-examined, resulting in a recall rate of 92% after 3 years. Compared to baseline, the 12-month and 24-month follow-up saw the loss of four patients. Three patients could not be relocated, one patient declined further participation. They were excluded (Figure 1).

### 3.3. Survival Rates after 36 Months

After 3 years the cumulative survival rate for all restorations was 95.7%. Four restorations failed. Compared to baseline, 12 months, and 24 months this means that another restoration failed in the last 12 months (Table 4). Every unsuccessful restoration, totaling 8.7%, was observed exclusively in the test group, with none occurring in the control group (0%). Consequently, the control group achieved a cumulative success rate of 100%, while the test group attained a rate of 91.3%. Regarding the annual failure rate, this resulted in significant difference between both groups: AFR of 0% in the control group and 2.9% in the test group (*p* < 0.05; Mann–Whitney U-test). 

### 3.4. Results of Parameters with Recognizable Findings after 36 Months

#### 3.4.1. Secondary Caries

After 36 months no secondary caries could be observed in any case. None of the 92 restorations placed in both groups showed any sign of secondary caries (control and test group, Table 4). 

#### 3.4.2. Tooth Vitality

Following the initial restorative procedure at baseline, three out of 92 teeth required non-surgical initial endodontic treatment (Code Delta) within the first 24 months and were rated as failures (Figure 1 and Table 4). All affected teeth, successfully endodontically treated, appertained to the test group. After 36 months recall no additional teeth lost their vitality. So, the remaining 89 included teeth reacted positively to the pulp vitality test (Table 4).

#### 3.4.3. Postoperative Sensitivity

The initially reported postoperative sensitivity immediately present after the restorative therapy could not be detected during the 3-year clinical examination. All teeth showed no signs of hypersensitivity and were rated as Code Alpha (Table 4).

#### 3.4.4. Filling Integrity/Fracture

Initially, after 12 months two restorations located in the test group showed a discreet chipping of the superficial composite material located at the distal margin (Code Bravo). One of the two teeth showed a continuous crack formation after 36 months and had to be rated as Code Charlie and considered as a failure (Table 4).

#### 3.4.5. Marginal Adaption

**Figure 2:**
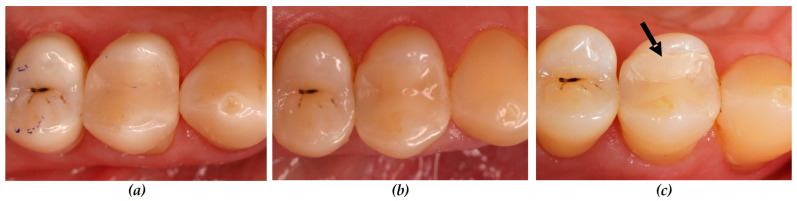
Clinical situation (Patient number 6): restoration tooth 14 (mod) without cavity lining: control group, (**a**) baseline: all criteria were rated Code Alpha; (**b**) after 12 months: all criteria rated as Code Alpha; (**c**) after 36 months: marginal adaption was rated as Code Bravo (arrow).

Initially, at baseline and after 12 and 24 months none of the initially applied restorations showed any findings regarding the parameter marginal adaption. After 36 months two restorations in the test group and one in the control group were rated as Code Bravo. (Figure 2, Table 4).

#### 3.4.6. Marginal Discoloration

**Figure 3:**
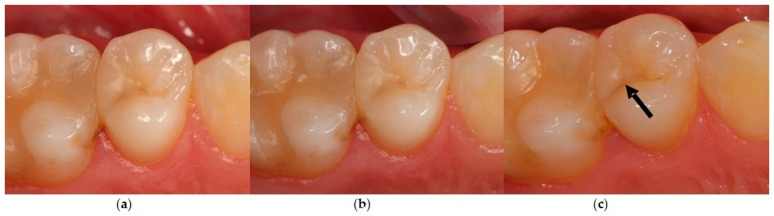
Clinical situation (Patient number 47): restoration tooth 15 (od) without cavity lining: control group, (**a**) baseline: all criteria were rated Code Alpha; (**b**) after 12 months: all criteria Code Alpha; (**c**) after 36 months: marginal discoloration was rated as Code Bravo (arrow).

**Figure 4:**
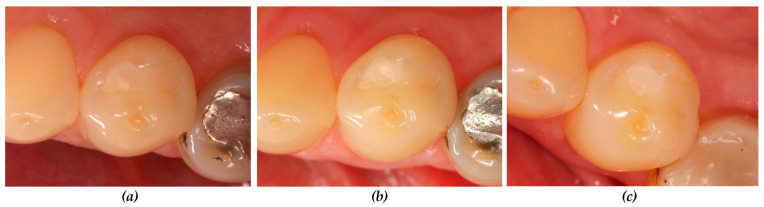
Clinical situation (Patient number 25): restoration tooth 34 (od) with cavity lining: test group, (**a**) baseline: all criteria were rated Code Alpha; (**b**) after 12 months: all criteria Code Alpha; (**c**) after 36 months: all criteria Code Alpha.

Already at the first re-examination appointment after 6 months, three restorations exhibited discolored restoration margins in two patients which were rated as Code Bravo. One restoration was located in the control and two in the test group. The number of affected fillings increased within the 2 years to five restorations in four patients showing superficial marginal discoloration (Code Bravo). Up to 36 months, this number increased to eight restorations revealing superficial marginal discoloration (Code Bravo). The difference between both groups was statistically significant (*p* < 0.05, Mann–Whitney U-test, Table 4). The control group (used without cavity lining) showed significantly more restorations with marginal discoloration compared to the test group (Figure 3 and Figure 4).

### 3.5. Results of Paramters without Any Findings after 36 Months

All other parameters—proximal contact, surface roughness, and color match—comparable to the 24 months interval, showed no findings in both groups after 3 years. In the case of the marginal adaption parameter, three restorations were rated as Code Bravo, one in the control and two in the test group (Table 4). Compared to the previously published 24 months results this means no significant increase of one restoration in both groups (*p* > 0.05, Mann–Whitney U-test).

### 3.6. Results of Oral Hygiene Indexes after 36 Months: Plaque and Gingival Index

After 36 months seven patients showed a PI of 1 and four patients exhibited a GI of code 1. After 3 years no significant differences were detectable regarding both the oral hygiene indexes PI and GI between both groups (*p* > 0.05, Mann–Whitney U-test). Compared to the baseline results (6 patients), the 6-months results (7 patients), the 12-months results (7 patients), and the 24-months results (5 patients) recorded a PI of 1, no further changes in oral hygiene deficiencies were visible after 36 months. Regarding the GI, in four patients a GI of 1 was found. This means the gingiva showed no inflammation, no bleeding but just a slight discoloration of the gingiva margin. Compared to the 24-months results there is an increase of two patients.

## 4. Discussion

After 36 months, 46 patients could be re-examined. At the beginning of the study, after screening 74 patients, 50 patients passed the inclusion and exclusion criteria and could be included. After 24 months, three patients had to be excluded and 47 could be re-evaluated. Two patients moved away and could not be contacted, and one patient refused further participation in the study [29]. At the present stage, after 36 months another patient had to be excluded because of leaving the region and not being able to attend further examinations (Figure 1). After 3 years 46 patients could be re-evaluated leading to a recall rate of 92% compared to 94% after 24 months, 100% after 12 months, and 100% after 6 months. Comparing this recall rate with other published studies focusing on the clinical success of restorative procedures over 3 years [26], the recall rate of 94% in the present investigation is quite high [38]. This might be an indicator for a carefully performed screening process including the exact medical and study information of the included patients. Taking the actual observation period of 36 months into account, the actual recall rate is comparably high and might allow evaluation of valid and representative results also with regard to the ongoing study over a longer period up to at least 48–60 months. Furthermore, the study protocol intended a randomized-controlled, single-blinded design, thus increasing internal validity [39,40].

Regarding the main aim of our clinical study, the overall cumulative survival rate, pooling both groups after 36 months regarding the successful codes (Alpha, Bravo), was 95.7%. Regarding both groups, the statistical analysis showed significant differences in the cumulative success rate of the control (100%) and test group (91.3%) within the observation period of 3 years. Comparing the present results with the results from a previously published clinical trial combining a regular and heavy flowable nanohybrid composite showed similar outcomes with no detectable Code Charlie or Delta ratings [32]. A clinical investigation by Ernst et al. [41] also showed similar results regarding the cavity lining group (92.8%) and the control group without any lining (94.6%). Their results are well in line with our findings after 3 years. Furthermore, other investigations described further clinical layering techniques, which might be useful to create highly successful and predictable restorations for our patients [42,43]. These publications, improving the clinical handling during the restorative procedure, are different from our cavity and dentin lining approach. In the present study a high-viscosity flowable composite was used to increase adaption to dentinal cavity walls and avoid higher polymerization stress. 

As recently published concerning the results after 24 months and after 36 months of observation, three teeth with restorations assigned to the test group underwent endodontic treatment between the 12- and 24-months recall appointment and were rated as Code Delta [29]. Unfortunately, in all cases endodontic treatment had to be initiated due to irreversible pulpitis. All of these teeth initially had deep caries lesions and therefore they all had in common that indirect pulp capping using calcium hydroxide was necessary. Following the study protocol, teeth with deep caries lesions and the need for indirect pulp-capping procedures were not excluded (Table 2). Therefore, it is remarkable that a considerable high number, 12 out of 92 teeth re-examined after 36 months, received indirect pulp capping [44,45]. Out of these 12 teeth, seven teeth were assigned to the control and five to the test group. After 3 years, beside the three teeth receiving endodontic treatment, the other nine teeth remained vital and showed a positive sensitivity. Furthermore, regarding the endodontic treated teeth, the composite restorations of these suffering teeth exhibited no signs of secondary caries or marginal failures. Taking this fact into account, it can be stated that the cavities were restored successfully. The circumstance that all endodontically treated teeth were located in the test group might be due to the initial randomization procedure at the beginning. However, following the study protocol, these teeth had to be rated as failures (Code Delta). Furthermore, one filling of the test group showed continuous occlusal crack formation and had to be rated as a failure (Code Charlie) after 36 months resulting in an overall AFR of 2.9%.

Focusing on parameters such as secondary caries, marginal adaption, postoperative sensitivity, color match, and surface roughness after 36 months of observation, all re-examined restorations showed acceptable clinical results (no Charlie and Delta scores, Table 4). 

Postoperative sensitivity is one possible complication after adhesive procedures and is discussed depending on the adhesive protocol used and the presence of internal and marginal leakage following polymerization stress and shrinkage [46,47,48]. Nevertheless, despite significant enhancements of commercially available dentin adhesive systems, the interface between dental hard tissues and the restoration materials remains the most vulnerable area within the originally established adhesive layer. Therefore, any malfunction, failure or complete loss of this interface results in a compromised internal or even marginal adaption followed by a subsequent retention loss of the restoration. The polymerization shrinkage of the used composite material depends on the composition, the filler type and amount, the degree of conversion during polymerization, the monomer content and finally on the polymerization time [45,49]. In the present paper, within the clinical procedure, each composite increment was light-cured for 30 s. Beside the improved properties of the high-viscosity flowable material used, this prolonged polymerization time might explain the improved outcome of the placed restorations in both groups.

Several in vitro studies have reported a decrease in marginal micro-leakage and internal cavities, along with an enhanced adaptation of the composite to dental hard tissues when utilizing low-viscosity, flowable composites as an intermediate cavity lining and stress-breaking layer [17,19,24,50]. However, it’s worth noting that contrasting findings exist, as some investigations did not identify positive effects on the marginal adaptation parameter [16,19,51]. However, clinical trials showed comparable results [25,52]. This is in accordance with our results. The benefit of cavity lining could not be shown after 3 years with regard to these parameters. 

In the case of marginal discoloration, a significant difference after 36 months of observation could be detected. Marginal discoloration was significantly more frequently evaluated in the control than in the test group combined with a flowable composite for cavity lining. In some recent studies it is known that preconditioning of the enamel with phosphoric acid results in better marginal integrity [53,54] and accordingly there is less marginal discoloration [54]. In our trial, all restorations in both groups were placed using the adhesive system in self-etch mode without prior conditioning. This could explain the comparatively high occurrence of marginal discoloration in both groups (10.9% in the control and 6.5% in the test group) in general, however, another control group to evaluate this point in which pretreatment with phosphoric acid was performed is missing. Currently, there are no studies that have investigated the influence of an intermediate liner in composite restorations on marginal discoloration. Concerning the marginal discoloration parameter, even higher scores were reported in analogical investigations. Regarding the study of Torres et al., evaluating the same composite material (Grandio^®^SO), Code Bravo was reported in 39.5% of the cases in the test group and 32.5% in the control group [32]. Further studies from Ernst et al., also using the same material, detected Code Bravo for 23.6% of the cases in the test and 32.1% in the control group [41]. It might be possible that the observable marginal discoloration could be the beginning of a debonding process, leading at the end to marginal discrepancies which could affect further parameters in subsequent prolonged follow-up evaluations. Regarding the self-etch adhesive system used in the present investigation, it is known from several published studies that bond strength to dentin is comparable between the etch-and-rinse and self-etch systems [55,56]. These results might explain the present findings. All restorations in both groups showed no increased rating scores in case of the marginal adaptation after 3 years. However, regarding other publications the formation of a marginal leakage and subsequently secondary caries is known to need more than 3 years [5,49,57,58,59], so prolonged long-term periods might be useful. Focusing on the use of flowable composites in adhesive dentistry, a remarkable number of in vitro studies describe an improved internal and marginal adaption [7,12,13]. On the other hand, numerous studies did not prove these positive findings [7,58,59]. Additionally to these in vitro studies the positive effect of flowable composite materials as cavity liner in posterior restorations compared to the application without using this lining technique has been investigated in comparable clinical studies [60,61]. Compared to most of the common flowable composite materials, the flowable resin material applied in our clinical long-term study is a so-called heavy flowable nanohybrid composite with a relatively high viscosity. Many investigations, evaluating the influence of lining with flowable composites on the clinical long-term success of direct composite restorations reported favorable results [32,43,62,63]. The present results using the high-viscosity flowable composite, which might be more effective when used as a liner in the posterior teeth requiring increased strength did not support these findings. After 3 years the estimated positive effects were not observable. Therefore, a prolonged observation period might show the improved quality of the restorations placed in combination with a high-viscosity flowable material. Considering the pivotal role of oral hygiene for caries development around composite restorations [61], patients initially displaying plaque accumulation were from the beginning actively encouraged to enhance their oral hygiene. At each appointment, a comprehensive professional dental cleaning, coupled with oral hygiene instructions, was administered. This might be the explanation for the relatively low levels of plaque accumulation and both associated indices at the 3-year follow-up. It’s crucial to acknowledge that individual subjects’ oral hygiene is a variable that may be influenced but not consistently controlled. To mitigate this aspect’s impact, the screening process and selection of study patients took into account the emphasis on good oral hygiene. The current findings from both indices align with the sustained absence of secondary caries after 3 years. Additionally, the clinical trial’s adoption of a split-mouth design serves to minimize individual differences related to caries risk factors such as oral hygiene deficiencies, saliva composition, and dietary habits [60,61,64,65,66]. Contrary to previous published investigations reporting the presence of secondary caries even two years after employing a flowable composite as a liner [41,67], the present investigation observed no instances of secondary caries. The thoroughly planned and established study protocol might be an explanation for these findings. However, a longer observation time might show different developments.

## 5. Conclusions

Initially, as already seen after 12 months of observation, three teeth receiving endodontic treatment due to vitality loss were rated as failures and one further restoration had to be rated as a failure after 3 years due to continuous crack formation in the test group used with the flowable cavity lining. Taking the endodontic origin of these failures into account, the nanohybrid composite material showed excellent clinical outcomes in posterior teeth over the observation period of 3 years. Regarding the impact of cavity lining, the results of the test group used with the high-viscosity flowable composite showed significantly increased annual failure rates (AFR) of 2.9% compared to 0% in the control group (*p* < 0.05; Mann–Whitney U test). Furthermore, except differences in tooth vitality, success rate, marginal discoloration, and AFR, no significant effects of the flowable composite on the other parameters were detectable. However, a prolonged observation time regarding several years might reveal more differences between both groups and could show an influence of flowable composites on restoration success. In conclusion, cavity lining with an additional layer of a high-viscosity flowable composite could be a clinical treatment possibility, because it might help dentists to improve the material adaption to the cavity walls and might reduce polymerization stress at the margins. 

## Figures and Tables

**Table 1:** The used inclusion and exclusion criteria.

Inclusion Criteria	Exclusion Criteria
adults (≥18 years) signed consent form available 2 molars/premolars with Class I/II cavity lesions with: - antagonist - approximal contact - bucco-oral extension at least one third of the spacing of tooth cusps positive vitality teeth needing indirect pulp capping	minors (<18 years) systemic diseases allergies related to materials pregnancy or breastfeeding inadequate oral hygiene preoperative pulpal symptoms endodontically treated teeth teeth needing direct pulp capping bruxism impossibility of absolute drainage by means of rubber dam patients who are not able to attend recall examinations

**Table 2:** Summary of the modified USPHS/Ryge criteria used for the clinical evaluation [29].

Modified USPHS/Ryge Criteria	Alpha	Bravo	Charlie	Delta
Secondary caries	No clinical signs of caries along the margin			Clinical diagnosis of caries
Tooth vitality	Positive			Negative
Postoperative sensitivity	No hypersensitivity	Minimal complaints only for a short timespan after placement, no treatment necessary	Medium complaints, no treatment necessary	Permanent complaints, bearable, treatment planned
Filling integrity/fracture	No chipping, cracking or fracturing of the filling	Chipping, crack formation, detectable with a probe	Continuous crack formation, visible	Bulk fracture of the restoration
Proximal contact	Tight approximal contact point	Slight approximal contact point	No approximal contact, no food impaction	No approximal contact point, trauma of papilla and food impaction
Surface roughness	Smooth, polished surface	Slightly rough surface, polishing is possible	Rough surface, polishing is not possible any longer	Fractured or flaking surface
Marginal adaption	No detectable margin (dental explorer)	Detectable margin without exposure of dentin or enamel	Visible margin with exposure of dentin or enamel	Fractured marginal interface, mobile, or lost restoration
Marginal discoloration	No marginal discoloration	Presence of superficial marginal discoloration	Presence of penetrating marginal discoloration	
Color match	Invisible restoration, perfect color match	Visible restoration without severe color mismatch	Clearly visible restoration with color mismatch in the normal range of tooth color	Highly visible restoration with color mismatch outside the normal range of tooth color

**Table 3:** Distribution and Black’s classification of dental lesions of the included study teeth.

	Premolar	Molar	Class I	Class II
Test group	20	30	20	30
Control group	22	28	12	38
Total number	42	58	32	68

**Table 4:** Summary of evaluated parameters according to the modified USPHS/Ryge criteria (Code Alpha/Bravo/Charlie/Delta) and Plaque index/Gingival index (Code 0/1/2/3): Examination at baseline, 24 months [29], and 36 months.

Parameter		Control Group			Test Group		
Interval		Baseline	24-Months Follow-Up	36-Months Follow-Up	Baseline	24-Months Follow-Up	36-Months Follow-Up
Biological properties	Secondary caries	50/0/0/0	47/0/0/0	46/0/0/0	50/0/0/0	47/0/0/0	46/0/0/0
	Tooth vitality	50/0/0/0	47/0/0/0	46/0/0/0	50/0/0/0	44/0/0/3	43/0/0/3
	Postoperative sensitivity	50/0/0/0	47/0/0/0	46/0/0/0	50/0/0/0	47/0/0/0	46/0/0/0
Functional properties	Filling integrity/fracture	50/0/0/0	47/0/0/0	46/0/0/0	50/0/0/0	45/2/0/0	44/1/1/0
	Proximal contact	50/0/0/0	47/0/0/0	46/0/0/0	50/0/0/0	47/0/0/0	46/0/0/0
	Surface roughness	50/0/0/0	47/0/0/0	46/0/0/0	50/0/0/0	47/0/0/0	46/0/0/0
	Marginal adaption	50/0/0/0	47/0/0/0	45/1/0/0	50/0/0/0	46/1/0/0	44/2/0/0
Aesthetic properties	Marginal discoloration	50/0/0/0	45/2/0/0	41/5/0/0	50/0/0/0	44/3/0/0	43/3/0/0
	Color match	50/0/0/0	47/0/0/0	46/0/0/0	50/0/0/0	47/0/0/0	46/0/0/0
Plaque index		44/6/0/0	42/5/0/0	39/7/0/0	44/6/0/0	42/5/0/0	39/7/0/0
Gingival index		50/0/0/0	45/2/0/0	42/4/0/0	50/0/0/0	45/2/0/0	42/4/0/0
n assessed		50	47	46	50	47	46
Recall rate (%)		100	94	92	100	94	92
n failure (cumulative failure %)		0 (0%)	0 (0%)	0 (0%)	0 (0%)	3 (6.8%)	4 (8.7%)
AFR (%)		0%	0%	0%	0%	3.4%	2.9%

## Data Availability

The data are available upon request from the corresponding author.
